# Multicomponent Self‐Assembly of a Giant Heterometallic Polyoxotungstate Supercluster with Antitumor Activity

**DOI:** 10.1002/anie.202017318

**Published:** 2021-04-09

**Authors:** Jian‐Cai Liu, Jie‐Fei Wang, Qing Han, Ping Shangguan, Lu‐Lu Liu, Li‐Juan Chen, Jun‐Wei Zhao, Carsten Streb, Yu‐Fei Song

**Affiliations:** ^1^ Henan Key Laboratory of Polyoxometalate Chemistry College of Chemistry and Chemical Engineering Henan University Kaifeng Henan 475004 China; ^2^ State Key Laboratory of Chemical Resource Engineering Beijing University of Chemical Technology Beijing 100029 China; ^3^ Henan-Macquarie University Joint Centre for Biomedical Innovation School of Life Sciences Henan University Kaifeng Henan 475004 China; ^4^ Institute of Inorganic Chemistry I Ulm University Albert-Einstein-Allee 11 89081 Ulm Germany

**Keywords:** bioactivity, nanostructures, self-assembly, supramolecular structures

## Abstract

The hierarchical aggregation of molecular nanostructures from multiple components is a grand synthetic challenge, which requires highly selective linkage control. We demonstrate how two orthogonal linkage groups, that is, organotin and lanthanide cations, can be used to drive the aggregation of a giant molecular metal oxide superstructure. The title compound {[(Sn(CH_3_)_2_)_2_O]_4_{[CeW_5_O_18_] [TeW_4_O_16_][CeSn(CH_3_)_2_]_4_[TeW_8_O_31_]_4_}_2_}^46−^ (**1 a**) features dimensions of ca. 2.2×2.3×3.4 nm^3^ and a molecular weight of ca. 25 kDa. Structural analysis shows the hierarchical aggregation from several independent subunits. Initial biomedical tests show that **1** features an inhibitory effect on the proliferation of HeLa cells based on an apoptosis pathway. In vivo experiments in mice reveal the antiproliferative activity of **1** and open new paths for further development of this new compound class.

## Graphical abstract

Hierarchical assembly of a giant heterometallic polyoxotungstate supercluster with a molecular weight of ca. 25 kDa is reported. Geometrically unrestricted cerium(III) and geometrically restricted dimethyl tin cation linkers are used to gain access to a giant molecular species featuring three different polyoxometalate building units. The compound demonstrates in vitro and in vivo antiproliferative activity against HeLa cervical cancer cell lines.

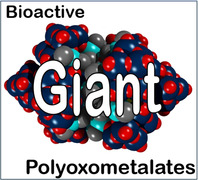

The controlled design of high‐nuclearity molecular metal oxides, so‐called giant polyoxometalates (POMs) is a cornerstone for bottom‐up access to nanoscale functional metal oxides.[^1^, ^2^, ^3^] In particular the assembly of hierarchically structured POM‐aggregates starting from well‐defined building units offers unique possibilities for bottom‐up materials design.[^4^, ^5^, ^6^] This approach therefore offers vast opportunities in fields ranging from biomedicine^[7]^ and molecular switches^[8]^ to energy conversion/ storage^[9]^ and quantum computing.^[10]^

Currently, the most common strategy for aggregating lacunary POM building units[^11^, ^12^] into large superstructures is the use of metal cation linkers such as transition‐metal (TM) or lanthanide (Ln) cations.^[13]^ Classic examples are based on tungstate Keggin‐ and Dawson‐anion derivatives, where assembly into giant supercluster aggregates[^14^, ^15^, ^16^, ^17^, ^18^, ^19^] or even POM‐based frameworks has been reported.[^2^, ^20^, ^21^] However, the control mechanisms which give access to giant molecular rather than infinite solid‐state structures are still not well understood. However, this understanding forms the basis for a knowledge‐driven POM materials design. Therefore, tremendous efforts have been dedicated to establishing and exploring the field of hierarchical POM superstructure assembly. One particularly promising approach has been the linkage of lacunary POMs using TM[^16^, ^22^, ^23^, ^24^, ^25^] or Ln[^14^, ^15^, ^18^, ^26^] linkages.^[13]^ However, the coordination behavior of most TM or Ln cations does not restrict growth and can result in the formation of 3D infinite frameworks.[^2^, ^20^, ^21^] A higher degree of aggregation control is possible by using site‐specific organotin and organoboron linkages.[^27^, ^28^, ^29^] This concept has been pioneered by Kortz and co‐workers, who explored the linkage of lacunary tungstate anions (**{W_9_}**) with dimethyl tin cations [Sn(CH_3_)_2_]^2+^ (**{Sn}**), resulting in tetrameric **{Sn}_3_{W_9_}_4_** ^[27]^ or even dodecameric **{Sn}_36_{W_9_}_12_** ^[28]^ superclusters. The authors showed that the two methyl groups of **{Sn}** effectively block coordination sites and thus prevent unrestricted growth of the aggregates into infinite frameworks. Building on this principle, recently, Chen, Streb and colleagues reported the hierarchical assembly of POM nanocapsules based on Dawson anions (**{M_3_W_15_}**, M=Nb, Ta) covalently linked by aromatic boronic acids (**{BA}**). The authors demonstrated that they can access tetrameric **{BA}_4_{M_3_W_15_}_4_** as well as dodecameric **{BA}_4_{M_3_W_15_}_4_** capsules where each **{BA}** covalently links up to three POMs via B‐O‐M bridges.^[29]^

Here, we hypothesized that the combination of geometry‐restricted lacunary polyoxotungstate anions and dimethyl tin cations with coordinatively more flexible Ln cations could give access to hierarchically structured supramolecular aggregates of POM clusters, leading to new structures and functions.^[30]^ This is inspired by pioneering studies which have demonstrated the use of tungstate superclusters in biomedicine,^[26]^ proton‐conduction^[19]^ and the design of molecular core–shell nanostructures.^[25]^ As an additional means of targeting larger, molecular POM aggregates, we opted for the incorporation of Te^IV^ based oxoanions as templates. This is based on previous studies which suggested that this approach can effectively prevent the uncontrolled coordination of multiple metal cations to one lacunary POM, which would either result in the formation of small POM‐clusters,^[12]^ or in infinite framework growth.^[2]^

We explored the self‐assembly of giant polyoxotungstate anions by combining geometry‐restricted [Sn(CH_3_)_2_]^2+^ cations and geometry‐unrestricted Ce^3+^‐cations together with tellurate‐templated lacunary tungstate clusters. Briefly, the target compound was obtained by reaction of ortho‐tungstate [WO_4_]^2−^ and tellurite [TeO_3_]^2−^ anions with Ce^3+^ and [Sn(CH_3_)_2_]^2+^ cations in aqueous solution in the presence of dimethylammonium NH_2_Me_2_^+^ cations (for synthetic details, see the Supporting Information). Single‐crystals suitable for X‐ray diffractometry (XRD) were obtained in yields of ca. 24 %. This enabled us to identify the title compound **1**, (NH_2_Me_2_)_22_K_12_Na_12_ [(Sn(CH_3_)_2_)_2_O]_4_{[CeW_5_O_18_][TeW_4_O_16_][CeSn(CH_3_)_2_]_4_[TeW_8_O_31_]_4_}_2_⋅ca.186 H_2_O (=(NH_2_Me_2_)_22_ K_12_Na_12_ **1 a**⋅ca.186 H_2_O). **1** crystallizes in the monoclinic space group *C*2/*c* with cell parameters *a=*35.818(2) Å, *b=*33.663(2) Å, *c=*50.865(4) Å, *β*=100.029(1)°, *V=*60 393(7) Å^3^ (Supporting Information, Table S1). **1** was fully characterized in the bulk by elemental analyses, thermal analysis and FT‐infrared spectroscopy (Supporting Information, Figure S1 and S2).

Structural analysis shows that the lattice of **1** features individual POM superclusters **1 a** with dimensions of ca. 2.3×2.3×3.4 nm^3^ (Figure 1 a). **1 a** is composed of 118 metal ions (82 W, 16 Sn, 10 Ce, 10 Te, Figure 1 b; Figure S3) with a molecular weight of ca. 25 kDa. To the best of our knowledge, **1 a** is the largest organotin‐ and Ln‐functionalized POM reported to date, and one of only three examples of this new compound class.[^31^, ^32^]

**Figure 1:**
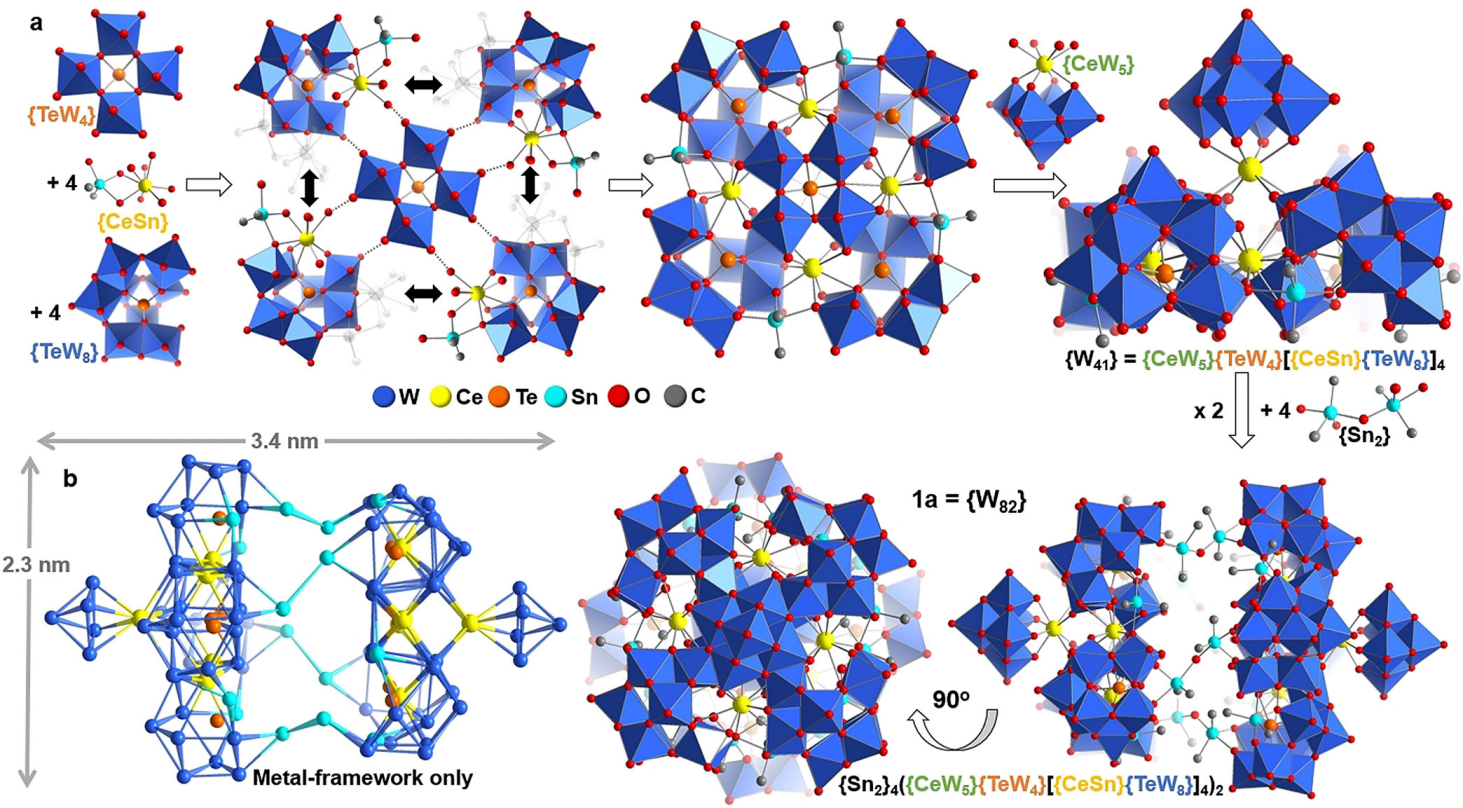
a) Schematic rationalization of the structure of **1**: one **{TeW_4_}** unit acts as an anchoring point for four **{TeW_8_}** clusters linked by four **{CeSn}** units. The resulting aggregate is capped by a **{CeW_5_}** Lindqvist cluster. Two aggregates are connected by four dinuclear **{Sn_2_}** units, giving the final cluster **1 a**=**{Sn_2_}_4_({CeW_5_}{TeW_4_}[{CeSn}{TeW_8_}]_4_)_2_**, dimensions ca. 2.3×2.3×3.4 nm^3^. b) Metal‐based skeleton of **1 a** showing the Ce, Sn, W and Te atoms only.

The structure of the large polyanion can be rationalized as follows: **1 a**, **{W_82_}** is a dimeric species composed of two principal, symmetry‐equivalent **{W_41_}** subunits. The **{W_41_}** units are assembled around a central *C*_4*v*_‐symmetric **{TeW_4_}** fragment, [TeW_4_O_20_]^12−^, where a central (rarely observed) square‐pyramidal [Te^IV^O_4_] links four [WO_6_] units in corner‐sharing mode (Figure 1 a; Figure S4). Around the **{TeW_4_}** fragment, four tetra‐vacant lacunary Keggin anions **{TeW_8_}** (=[B‐α‐TeW_8_O_31_]^10−^)^[31]^ are assembled. The aggregate is linked by four Ce^3+^ cations and four Sn(CH_3_)_2_^+^ groups, each forming three Sn‐O‐*M* bonds (M=Ce, W), resulting in a **{CeSn}** linkage (Figure 1 a; Figures S5–S9). Essentially, the **{TeW_8_}** units are linked around the central **{TeW_4_}** in a “head‐to‐tail” fashion, resulting in a large aggregate **{TeW_4_}[{CeSn}{TeW_8_}]_4_** (Figure 1). The aggregate‐face opposite the **{TeW_4_}** unit is further shielded from the environment by a cerium‐substituted lacunary Lindqvist anion **{CeW_5_}** (=[CeW_5_O_18_]^3−^, Figure 1 a). The **{CeW_5_}** forms one Ce‐O‐W coordinative bond to each **{TeW_8_}** unit, thereby providing further structural reinforcement (Figure S5).^[33]^ In sum, the **{W_41_}** subunit (=**{CeW_5_}{TeW_4_}[{CeSn}{TeW_8_}]_4_**) is obtained. The full cluster anion **1 a** (**{W_82_}**=**{W_41_}_2_**) is formed by linkage of two identical **{W_41_}** units by four dinuclear **{Sn_2_}** (=[(Sn(CH_3_)_2_)_2_O]^2+^) groups. Each **{Sn_2_}** bridges between two **{TeW_8_}** clusters in opposite **{W_41_}** subunits via Sn‐O‐W bonds, resulting in a torsion angle between the two **{W_41_}** units of ca. 45° (Figures 1 and 2). In sum, the giant polyanion **1 a** is hierarchically self‐assembled from three different lacunary tungstates; each unit fulfils a specific structural role: **{TeW_4_}** acts as central template and anchor, **{TeW_8_}** is a ditopic linkage group and **{CeW_5_}**, acts as monotopic capping site (see the simplified depiction in Figure 2).

**Figure 2:**
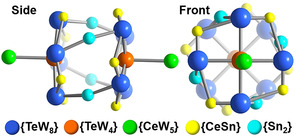
Simplified structure of **1 a** in front and side view, highlighting the hierarchical aggregation of three different lacunary POM building units **{TeW_4_}**, **{TeW_8_}** and **{CeW_5_}**, as well as the **{CeSn}** and **{Sn_2_}** linkages.

Next, we hypothesized that **1 a** could feature bioactivity; this is based on earlier reports, which demonstrated that POMs can act as artificial enzymes, and can exhibit antibacterial, antiviral and antitumor properties.[^7^, ^34^, ^35^, ^36^, ^37^] In particular, a range of polyoxotungstates (some of which featuring organotin functions)[^38^, ^39^, ^40^] have been reported as antitumor agents with possible use in cancer treatment.[^7^, ^34^, ^35^, ^36^, ^37^] Further, the ability to also incorporate lanthanides could in future enable simultaneous distribution analysis, for example, by magnetic resonance imaging (MRI).^[41]^ To date, antitumor studies of POMs have almost exclusively been focused on small POM derivatives, while to‐date, the use of giant POMs as anticancer treatments is still in its infancy.^[26]^ Based on this observation, we hypothesized that the diverging structures and properties of giant POMs could significantly affect their function as antitumor agents.

Initial cytotoxicity tests against human cervical cancer (HeLa) cells were performed to gain further insights into the reactivity of **1** in vitro. First, the stability of **1** in water, PBS, Serum and DMEM was assessed by time‐dependent FT‐IR and Raman spectroscopy spectra and Raman spectra (Figures S10 and S11), and no changes of the characteristic cluster‐signals were observed. However, note that while these studies suggest stability of **1** under the experimental conditions, they do not allow prediction of the behavior of the cluster when taken up by cells, as interactions with cell components or other biomolecules cannot be predicted at this point.[^7^, ^34^]

Next, cytotoxicity was assessed using colorimetric MTT assays after HeLa cells were incubated with **1** (0.01, 0.1, 1.0, 10.0 and 100.0 μM) for 24 h. MTT results indicate that the viability of HeLa cells decreases to 19.4 % [**1**]=100 μM. In contrast, all precursor reference compounds (Na_2_WO_4_⋅2 H_2_O, K_2_TeO_3_, Ce(NO_3_)_3_⋅6 H_2_O and Sn(CH_3_)_2_Cl_2_), as well as the commercially available 5‐fluorouracil (5‐FU) studied show significantly higher HeLa viabilities at identical concentrations of 100 μM (Na_2_WO_4_⋅2 H_2_O: 84.7 %; K_2_TeO_3_: 43.0 %, Ce(NO_3_)_3_⋅6 H_2_O: 79.0 %; Sn(CH_3_)_2_Cl_2_: 90.9 %; 5‐FU: 79.94 %), thereby highlighting the higher inhibitory performance of **1** against HeLa proliferation (Figures S12 and S13). Based on these data, **1** features an IC_50_ value of 5.65±0.83 μM against HeLa cells (Figures S14 and S15). In addition, **1** also shows good cytotoxicity against human breast cancer (MCF‐7) cells, human hepato‐cellular carcinoma (HepG2) cells, human lung adenocarcinoma (A549) cells, human liver (SMMC‐7721) cells and murine melanoma (B16F17) cells (Figure S16). MTT results reveal that **1** is also cytotoxic toward kidney epithelial cells (Figure S17), thus highlighting that introducing cancer‐specific targeting, for example, using bioconjugation, will be key requirement for the further development of this compound class.^[42]^

We further examined the uptake of **1** by HeLa cells. Inductively coupled plasma optical emission spectrometry (ICP‐OES, Figure S18) as well as confocal fluorescence spectroscopy and UV/Vis spectroscopy (Figures S19–S21), showed, that the POM is taken up by HeLa cells in a time‐ and concentration‐dependent fashion. Biotransmission electron microscopy (TEM) images and elemental mapping further demonstrate the presence of the relevant elements (W, Te. Sn) in the HeLa cells (Figure S22).

Next, we performed initial mechanistic studies to understand the antitumor mode of action of **1**, as we hypothesized that **1** might induce cell apoptosis rather than uncontrolled necrosis.^[43]^ This was evaluated by flow cytometry (annexin V‐FITC and propidium (PI) assays), analysis of mitochondrial membrane potentials (MMP), Western Blot (WB) analysis and terminal deoxynucleotidyl transferase‐mediated dUTP nick‐end labelling (TUNEL) assays.[^44^, ^45^] As shown in Figure 3 a and Figure S23, HeLa cell apoptosis shows a clear time‐dependent behavior, and ca. 54.8 % apoptotic cells are observed after incubation with **1** for 48 h. In contrast, less than 10 % of the untreated cells undergo apoptosis within 48 h, indicating that **1** induces the apoptosis of HeLa cells. This time‐dependent behavior of **1** is supported by MMP assays (Figure 3 b; Figure S24) using JC‐1 cationic fluorescent dye labelling to assess cell viability.^[46]^

**Figure 3:**
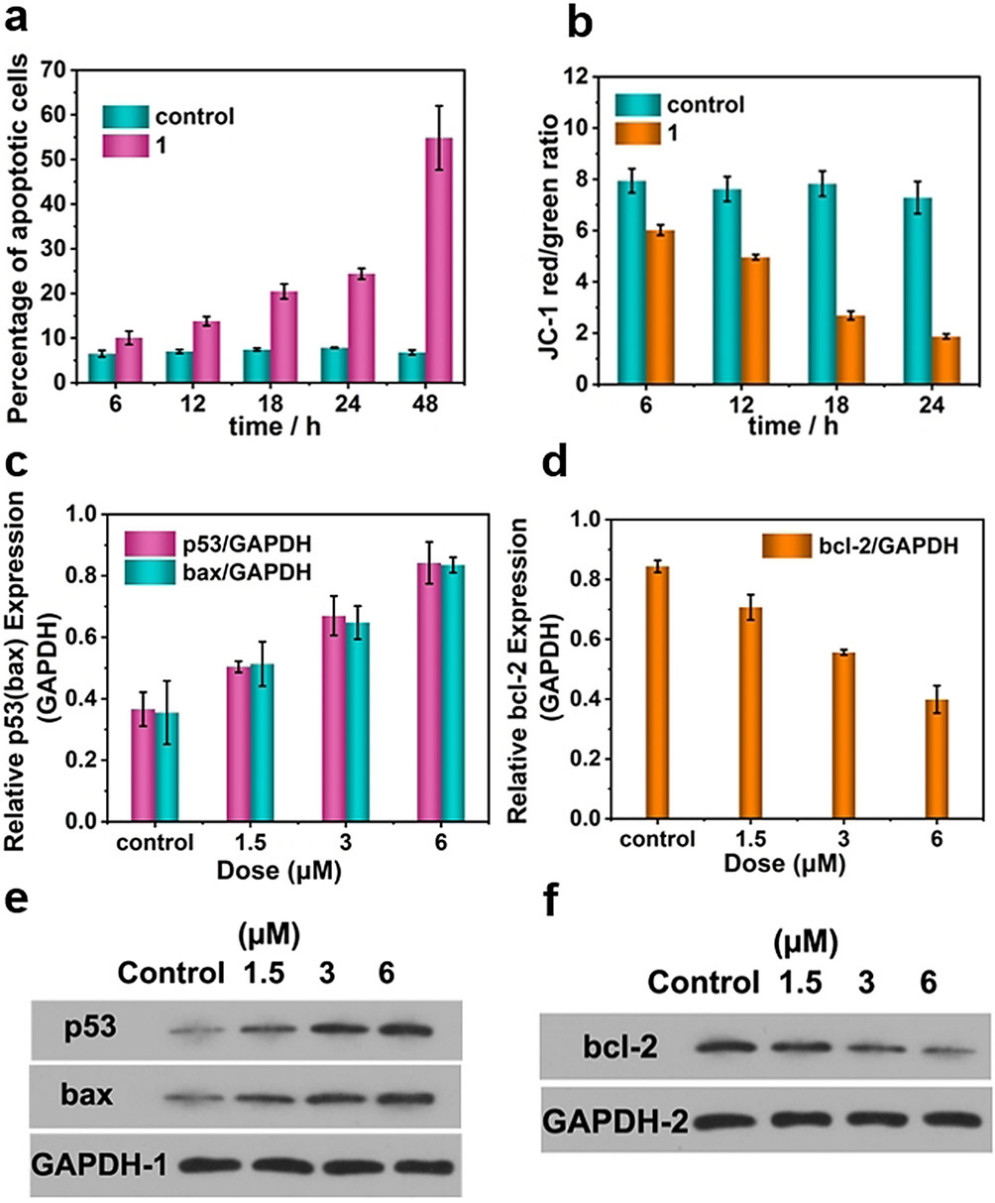
a) Flow cytometry analysis of apoptosis and statistical results. Cells were treated with 3 μM **1** for 6 h, 18 h, 24 h and 48 h and stained with Annexin V‐FITC‐PI. b) Effect of **1** (3 μM) on the MMP in HeLa cells with various durations of 6 h, 12 h, 18 h and 24 h. c–f) Expression of p53 (c,e), bax (c,e) and (d,f) bcl‐2 in HeLa cells determined by WB analyses. GAPDH was used as the loading control. The cells were treated with **1** with variable concentrations. The data are presented as mean ± SD based on three replications.

Next, we used WB analyses to gain further understanding of the function of **1**. To this end we analyzed the expression marker proteins p53/bax (pro‐apototic) and bcl‐2 (anti‐apoptotic) which play a key role in apoptosis pathways and tumor growth.^[47]^ Our data indicate a positive correlation between the expression of bax and p53 proteins and the concentration of **1** (Figure 3 c,e), while the expression of bcl‐2 is down‐regulated at increasing concentration of **1** (Figure 3 d,f). This suggests that **1** triggers cell apoptosis by suppressing bcl‐2 expression and accelerating p53/bax expression.

Further insights were provided by the TUNEL assay, which identifies DNA fragments formed by apoptosis.^[48]^ Over time, we observe a continuous increase of apoptotic cells (Figures S25 and S26). After 48 h, the apoptosis index (i.e. the ratio of apoptotic to total cells) is >50 %. In contrast, the apoptosis indices of the reference non‐treated cell samples (3.7 %–4.8 %) shows only minor changes over 48 h. In sum, these combined studies demonstrate that **1** can induce cell apoptosis in cancerous HeLa cell lines.

Finally, initial in vivo experiments were carried out to study the antitumor activity and toxicity of **1** using HeLa‐bearing mice as model organisms. As treatment, one subgroup of mice were injected once daily with saline solutions of **1** (100 mg kg^−1^) for 10 days, while a control group was kept for reference. This first study shows that tumor progression was significantly inhibited based on comparison of the tumor volume (at day 18). For the **1**‐treated mice, an average tumor volume of 677 mm^3^ was observed, while the control group showed an average volume of 1247 mm^3^ (Figure S27). Note that the body weight of the mice did not show obvious changes, suggesting no major side effects of **1** (Figure S27). In addition, WB analyses show that treatment with **1** significantly increases the expressions of p53/bax and decreases the expression of bcl‐2 (Figure S28), while hematoxylin and eosin (H&E) staining reveals the appearance of inflammation lesions and local necrosis for **1**‐treated tumors (Figure S29). In the control group, no tumor damage was evident. Finally, TUNEL assays show a higher number of apoptotic cells for the **1**‐treated tumors compared with the controls (Figure S29). In sum, the results suggest that **1** can suppress tumor growth by inducing cell apoptosis.

In summary, we report a facile self‐assembly‐based bottom‐up route which gives access to hierarchically structured giant polyoxometalate superclusters. The synthetic concept is based on the use of lacunary POM together with two orthogonal metal cation linkages (i.e., geometrically restricted dimethyl tin and geometrically unrestricted cerium(III) cations). This combination results in the assembly of a multicomponent nanosized molecular aggregate, where three distinct lacunary POM classes are combined in one giant species. Initial studies highlight that the compound combines promising antitumor activity against HeLa cancer cells with low general cytotoxicity. To our knowledge, this is the first reported example of a giant organotin/lanthanide functionalized POM supercluster with antitumor activity. In future, we aim to expand this novel compound class to further explore biological and biomedical functions.

## Conflict of interest

The authors declare no conflict of interest.

## Supporting information

As a service to our authors and readers, this journal provides supporting information supplied by the authors. Such materials are peer reviewed and may be re‐organized for online delivery, but are not copy‐edited or typeset. Technical support issues arising from supporting information (other than missing files) should be addressed to the authors.

Supplementary
