# Supplementary material for: Multicomponent Self‐Assembly of a Giant Heterometallic Polyoxotungstate Supercluster with Antitumor Activity
Source: Angew Chem Int Ed Engl. 2021 Apr 9;60(20):11153–7. doi: 10.1002/anie.202017318 (PMC8252014; doi:10.1002/anie.202017318)
Supplement: Supplementary file 1 — Supplementary [file ANIE-60-11153-s001.pdf]

Angewandte Chemie
Eine Zeitschrift der Gesellschaft Deutscher Chemiker

Supporting Information

Multicomponent Self-Assembly of a Giant Heterometallic Polyoxotungstate Supercluster with Antitumor Activity

Jian-Cai Liu, Jie-Fei Wang, Qing Han, Ping Shangguan, Lu-Lu Liu, Li-Juan Chen, Jun-Wei Zhao, Carsten Streb,* and Yu-Fei Song**

anie_202017318_sm_miscellaneous_information.pdf

Supporting Information

©Wiley-VCH 2021
69451 Weinheim, Germany

DOI: 10.1002/anie.2020XXXXX

Table of Contents

S1 Experimental procedures 2

S2 X-ray crystallography 3

S3 Additional figures 4

S4 Bond valence sum (BVS) calculations 19

S5 References 19

S6 Author Contributions .... **Fehler! Textmarke nicht definiert.**

S1 Experimental procedures

1.1 Materials and Measurements

All chemicals and reagents were purchased commercially and used without further purification. IR spectra were obtained using KBr pellets on a Nicolet 170 SXFT-IR spectrophotometer in the wavenumber range between 4000 to 450 cm^{-1}. C, H and N elemental analyses were conducted on a Perkin-Elmer 240C elemental analyzer. Inductively coupled plasma atomic emission spectrometry (ICP-AES) were performed on a Perkin−Elmer Optima 2000 ICP-AES spectrometer. Thermogravimetric (TG) analysis was carried out on a Mettler–Toledo TGA/SDTA 851e instrument under a flow of N_2 with a heating rate of 10 °C / min from 25 to 1000 °C. Photoluminescence spectra were measured on an Edinburgh FLS 980 analytical instrument equipped with a 450 W xenon lamp. Raman spectra were recorded on an Edinburgh RM5 Raman spectrometer with an excitation laser at 530 nm. Confocal laser scanning microscopy images were obtained on a Zeiss 880 microscope. The morphologies and elemental mappings of the microtome sections of fixed cells were observed using a transmission electron microscope (TEM, JEOL JEM-F200).

1.2 Synthesis of 1

$Na_2WO_4{\cdot}2H_2O$ (2.002 g, 6.069 mmol), K_2TeO_3 (0.201 g, 0.792 mmol), 1,3-bis(tris(hydroxymethyl)methylamino)propane (0.301 g, 1.066 mmol) and dimethylamine hydrochloride (1.002 g, 12.288 mmol) were dissolved in 30 mL distilled water under stirring. Glacial acetic acid (0.5 mL, 8.743 mmol), $Ce(NO_3)_3{\cdot}6H_2O$ (0.251 g, 0.578 mmol) and $(CH_3)_2SnCl_2$ (0.252 g, 1.147 mmol) were consecutively added to the solution and the final pH of the solution was kept at 5.6 using 6.0 M aqueous HCl. This solution was stirred for 10 min, heated at 90 °C for 2 h, cooled to room temperature and filtered. The filtrate was left to evaporate for crystallization. Yellow block crystals of **1** were isolated after three or four weeks. Yield: 0.42 g (24.1 % based on Ce). Anal. Calcd (Found %) for $C_{76}H_{644}Ce_{10}K_{12}N_{22}Na_{12}O_{506}Sn_{16}Te_{10}W_{82}$: C, 3.01 (3.27); H, 2.14 (2.31); N, 1.01 (1.16); K, 1.55 (1.75); Na, 0.91 (1.32); Sn, 6.26 (6.74); W, 49.65 (50.19); Ce, 4.61 (4.45); Te, 4.20 (4.43).

1.3 Anticancer experiments

Cell culture. The HeLa (human cervical cancer) cells, MCF–7 (human breast cancer), HepG2 (human hepatocellular carcinoma), A549 (human lung adenocarcinoma), SMMC-7721 (human liver) cells and B16F17 (murine melanoma) cells were purchased from Chinese Academy of Sciences. HeLa cells, MCF–7, A549 and B16F17 cells were maintained in DMEM (dulbecco′s modified eagle medium) supplemented with 10 % FBS (fetal bovine serum) and 1% penicillin-streptomycin solution. 293 cells were cultured in DMEM supplemented with 10 % FBS. HepG2 and SMMC-7721 cells were maintained in 1640 medium supplemented with 10 % FBS and 1% penicillin-streptomycin solution. All cells were cultured under CO_2 (5 %) atmosphere at 37 °C.

Cell proliferation. The cell viability was evaluated by MTT (3-(4,5-dimethylthiazol-2-yl)-2,5-diphenylte-trazolium bromide) experiments. Cells were placed in a 96-well plate at a density of 5×10^4 cells/mL cultured in DMEM supplied with 10% FBS + 1% penicillin-streptomycin solution, and each well plate has 100 μL cell suspensions. After 24 h of incubation, the cells were treated with various concentrations (0.01, 0.1, 1, 10, 100 μM) of **1** (dissolved in distilled water), and the reference compounds $Na_2WO_4{\cdot}2H_2O$, $Ce(NO_3)_3{\cdot}6H_2O$, $Sn(CH_3)_2Cl_2$ and K_2TeO_3 for 24 h. Each experiment at each concentration was repeated three times. Then cells continued to be incubated with the addition of 10 μL MTT per well for 4 h at 37 °C. After the removal of medium, 150 μL DMSO (dimethyl sulfoxide) was added to each well and the plate was shaken for 10 min. Absorbance spectrophotometry was measured at 568 nm using a microplate reader (MULTISKAN MK3, Thermo). The cell inhibitory rate with different concentrations was calculated. Cytotoxicity tests for MCF–7, A549, HepG2, B16F17, SMMC-7721 and 293 cells were conducted in the same manner. IC50 values of **1**, $Na_2WO_4{\cdot}2H_2O$, $Ce(NO_3)_3{\cdot}6H_2O$, $Sn(CH_3)_2Cl_2$ and K_2TeO_3 against HeLa cells were evaluated based on a more restricted concentration range: for **1**, the concentrations are 2, 4, 5, 7 and 9 μM, respectively; for $Na_2WO_4{\cdot}2H_2O$, the concentrations are 400, 600, 800, 1000 and 1200 μM, respectively; for $Ce(NO_3)_3{\cdot}6H_2O$, the concentrations are 200, 400, 500, 600 and 800 μM, respectively; for $Sn(CH_3)_2Cl_2$, the concentrations are 200, 400, 600, 800 and 1000 μM, respectively; for K_2TeO_3, the concentrations are 20, 40, 50, 70, 90 μM, respectively. The MTT of 5-FU toward HeLa cells has been performed by treating with different concentrations of 5-FU (0.01, 0.1, 1, 5, 10, 100, 400, 600 and 800 μM). The drug-free experiments were carried out simultaneously.

Flow cytometry analysis of cell apoptosis. The HeLa cells were cultured on a 6-well plate at a density of $5{\times}10^5$ cells per well and incubated at 37 °C for 24 h. Then 3 μM **1** (dissolved in distilled water) was respectively added for 6, 12, 18 and 24 h, collected and washed

twice with PBS (phosphate buffer saline). The harvested cells were resuspended in 500 μL binding buffer. Subsequently, 5 μL Annexin V-FITC and 5 μL PI (propidium iodide) were added successively to the suspension. The cells were incubated in the dark for 5–15 min at ambient temperature, and analyzed utilizing a flow cytometer (CytoFLEX). The drug-free experiments were carried out simultaneously under the same conditions. Negative control experiments (Neither Annexin V-FITC nor PI were added to the solvent containing normal cells) and positive control experiments (only 5 μL Annexin V-FITC was added to the solvent where apoptosis in HeLa cells was the most obvious; only 5 μL PI was added to the solvent where apoptosis in HeLa cells was the most obvious) were performed.

Measurements of mitochondrial membrane potential (MMP). MMP was measured by using a lipophilic dye JC-1 (5,5,6,6-tetrachloro-1,1,3,3-tetraethylbenzamidazolocarbocyanin iodide). The HeLa cells were cultured on a 6-well plate at a density of 5×10^5 cells per well and incubated at 37 °C for 24 h. Then 3 μM **1** (dissolved in distilled water) was respectively added for 6 h, 12 h, 18 h and 24 h, collected and resuspended in 0.5 mL DEME. The drug-free experiments were carried out simultaneously under identical conditions. Cells with different treatments were incubated with JC-1 (0.5 mL) at 37 °C for 20 min in the incubator. After incubation, cells were centrifuged (1200 rmp, 4 °C, 3 min), collected and washed twice with JC-1 (1X) [JC-1 (1X) was prepared by adding 4 mL distilled water to 1 mL JC-1 (5X)]. Finally, cells were resuspended in 500 μL JC-1 (1X), and analyzed by flow cytometry. The ratio of fluorescence represents the change on MMP.

Western blotting (WB). After incubation with **1** (1.5 μM, 3 μM and 6 μM; dissolved in distilled water) in DMEM supplied with 10% FBS + 1% penicillin-streptomycin solution for 24 h, the total protein of the HeLa cells was extracted in the lysis buffer based on a published procedure.[1] The total protein (40 μg) was separated by a 5 % SDS–polyacrylamide gel, and then transferred to the PVDF membranes. Next, the membranes were immersed in blocking buffer containing 5 % non-fat dry milk for 2 h at room temperature. After blocking, the membranes were incubated with the primary antibodies of bax, p53 and bcl-2 in blocking buffer overnight at 4 °C. The membranes were washed five or six times for 5 min per time by TBST, and then incubated with the corresponding IgG HRP conjugate secondary antibody for 2 h at 37 °C. The membranes were washed five or six times for 5 min per time by TBST again to remove excessive secondary antibody. GAPDH protein was utilized as loading control. Protein bands were visualized and detected by the ChemiDoc XRS chemiluminescence detection and imaging system (Tianjin hanzhong photography material factory).

***In vivo* experiments**. Fifteen 4–6 week-old female nude mice with weight of 17–23 g were purchased from Liaoning province and used for the *in vivo* experiments. All animal experiments were conducted based to the guidelines outlined in the "Guide for the Care and Use of Laboratory Animals", and the Biomedical Research Ethics Sub-Committee of Henan University reviewed and approved the experimental protocols (approval no: 13937884027). The mice were divided into 2 groups and each group had 5 samples. The subcutaneous injection model is established in the back of the mice with the treatment of HeLa cells (8×10^6 cells / one mouse) after careful treatment for one week. The tumor volume (V) was determined by the method: $V = 1/2 \times a \times b^2$, where a and b represents the long and short diameter of tumor, respectively. These dimensions were determined by vernier caliper. When the tumor sizes grow to 70 mm^3 after tumor inoculation, one group of mice were treated with **1** (dissolved in saline water) at a dose of 100 mg/kg based on the weight of mice once every day for a total of 10 days. In addition, another group of mice were left untreated as reference individuals. The tumor volume and body weight of each mouse were carefully recorded every day. All mice were sacrificed on the eleventh day, and the excised tumors were weighted and divided into two parts, one fixed with 4% paraformaldehyde and one preserved at T= –80 °C for Western blotting, H&E staining and TUNEL assay.

In-vitro fluorescence imaging. HeLa cells were seeded on acid-treated, sterile coverslips placed in 24-well plates. After HeLa cells were incubated with **1** (100 μM) for 12 h, the cells were rinsed three times with PBS, and then fixed in newly prepared paraformaldehyde for observation using a confocal fluorescence microscope with 488 nm laser excitation.

Statistical analysis. Data were obtained as means ± standard deviation (SD). Values of $P < 0.05$ were considered as statistically significant.

S2 X-ray crystallography

A suitable single crystal of **1** was picked and sealed in a glass tube. Intensity data collection were carried out on a Bruker APEX−II CCD sealed tube diffractometer with graphite monochromated Mo Kα radiation (λ = 0.71073 Å) operating at 2986 K. The SADABS program was utilized for absorption correction.[2] The structure was solved by direct methods and refined by full-matrix least-squares on F^2 using the SHELXTL-97 program package.[3] In the refinement, the W, Te, Ce, Sn, K and Na atoms were refined anisotropically

while the C, N and O atoms were refined isotropically. Where possible, water molecules and dimethylammonium cations were located from the Fourier map. In addition, 12 Na^+ ions, 4 K^+ ions, 14 $NH_2Me_2^+$ ions and 134 water molecules were directly added to the molecular formula based on the elemental analyses and TG data. Crystallographic data and structure refinements for **1** are listed in Table S1. Crystallographic data of **1** have been deposited in the Cambridge Crystallographic Data Centre with CSD 2040588. These data can be obtained from The Cambridge Crystallographic Data Centre via www.ccdc.cam.ac.uk/data_request/cif.

Table S1. A summary of crystal data and structural refinements of **1**.

	1
Empirical formula	$C_{76}H_{644}Ce_{10}K_{12}N_{22}Na_{12}O_{506}Sn_{16}Te_{10}W_{82}$
CCDC code	2040588
Formula weight	30169.33
Crystal system	monoclinic
Space group	*C*2/c
T (K)	296
a (Å)	35.818(2)
b (Å)	33.6632(18)
c (Å)	50.865(4)
α(°)	90.00
β(°)	100.0290(10)
γ(°)	90.00
V(Å^3)	60393(7)
Z	4
D_c (Mg·m^{-3})	3.318
μ (mm^{-1})	17.528
F(000)	54117
θ limits (º)	1.63 to 25.00
Reflections collected / unique	144532 / 51962 [R_{int} = 0.1380]
Data / restraints / parameters	51962 / 283 / 2317
GOOF	1.053
R index [$I > 2\sigma(I)$]	R_1 = 0.0754, wR_2 = 0.1452
R (all data)	R_1 = 0.1932, wR_2 = 0.1682

S3 Additional figures

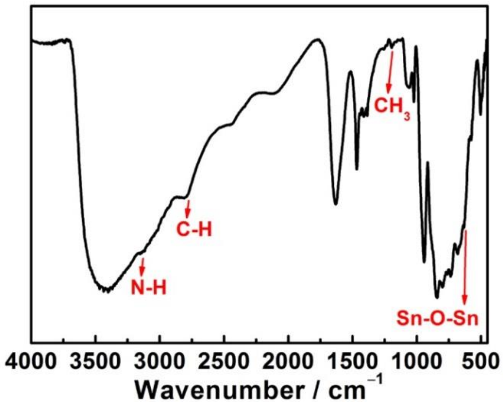

Figure S1. FT-IR spectrum of **1**.

As shown in Figure S1, three groups of characteristic peaks at 942; 847, 805, 762; and 683 cm^{-1} for **1** are assigned to the v(W–O_t), v(W–O_b), and v(W–O_c) stretching vibrations, respectively. The v(Te–O_a) stretching vibration peak is observed at 737 cm^{-1}. The weak peak at 1195 cm^{-1} is characteristic of the bending vibration of $\delta_s(CH_3)$ in $[(CH_3)_2Sn]^{2+}$ groups.[4–5] The peaks at 3139 and 2795 cm^{-1} are

respectively assigned to the N−H and C−H stretching vibrations, indicating the presence of monprotonated dimethylamine cations. The band centered at 633 cm^{-1} can be ascribed to the *v*(Sn–O–Sn) vibration,[6] in good accordance with the $[Sn(CH_3)_2O]$ groups in **1**. Besides, the broad bands appearing at 3414–3435 cm^{-1} as well as the intense bands at 1631 cm^{-1} correspond to the typical stretching and bending vibrations of water molecules.

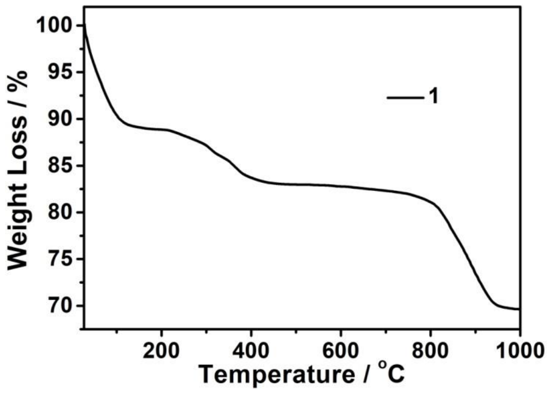

Figure S2. The TG curve of **1**.

As shown in Figure S2, the TG curve of **1** displays a three-step weight loss procedure. The first weight loss of 11.1 % (calcd 11.0 %) from 25 to 190 °C is based on the release of 186 lattice water molecules. The second weight loss step of 6.6 % (calcd. 6.3 %) from 190 to 700 °C corresponds to the release of the organic combustible components (22 $NH_2Me_2^+$, 32 methyl groups (from $Sn(CH_3)_2^+$) and 8 O groups from **{Sn2}**). At temperatures > 700 °C, further degradation of **1** is observed, weight losses correspond to sublimation of metal oxo species.

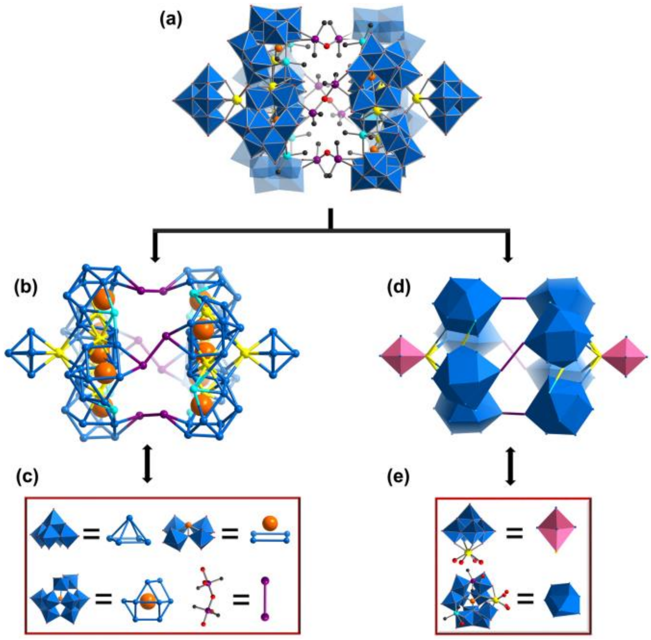

Figure S3. (a) Combined polyhedral/ball-and-stick view of **1**. The Sn atoms in the $\{[Sn(CH_3)_2]_2O\}^{2+}$ dimer and the $\{(TeW_4)Sn_4Ce_4(TeW_8)_4\}$ unit are distinguished in violet and turquoise color, respectively. (b)–(c) The metal-based skeleton of **1**. (d)–(e) Simplified model of **1**.

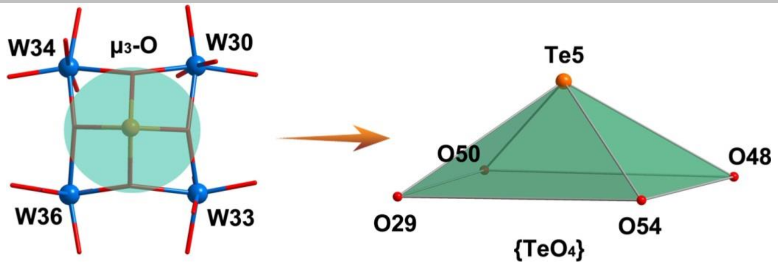

Figure S4. The $\{TeW_4\}$ core and the tetra-coordinate square-pyramidal environment of the $Te5^{IV}$ atom.

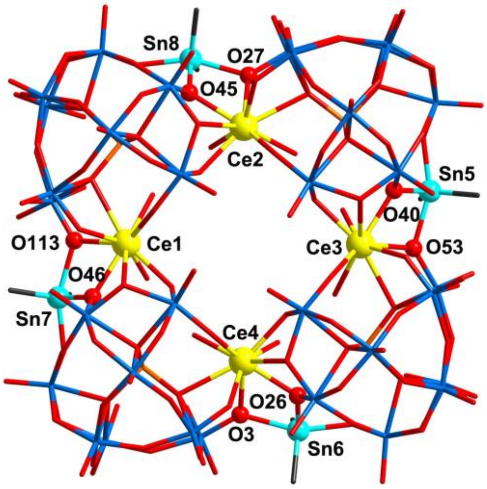

Figure S5. The connection of four $\{TeW_8\}$ units by Ce^{3+} and $[(CH_3)_2Sn]^{2+}$ linkers, as well as the connection of the Ce^{3+} linker in one $\{TeW_8\}$ unit with the $[(CH_3)_2Sn]^{2+}$ linker in another $\{TeW_8\}$ unit by sharing O atoms.

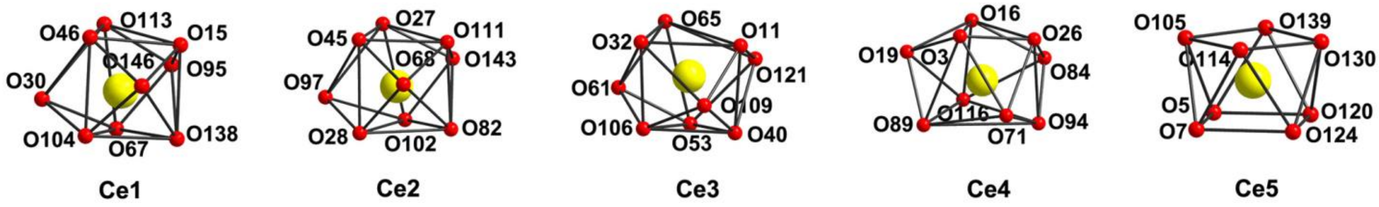

Figure S6. The coordination spheres and coordination geometries of Ce^{3+} ions in **1**.

There exist five crystallographically independent Ce^{3+} ions in **1**. $Ce1^{3+}$–$Ce4^{3+}$ ions are nona-coordinate forming distorted tri-capped trigonal prismatic geometry constructed from seven O atoms from two $\{TeW_8\}$ moieties and two O atoms from the $\{TeW_4\}$ section. The Ce–O distances are in the range of 2.36 (2)–2.883 (15) Å (Ce1), 2.373 (17)–2.846 (15) Å (Ce2), 2.392 (17)–2.841 (15) Å (Ce3), 2.377 (19)–2.854 (15) Å (Ce4), respectively. Ce5 ion exhibits the octa-coordinate distorted square antiprismatic environment defined by four terminal O atoms from one Lindqvist $[W_5O_{18}]^{6-}$ fragment and four O atoms from four $\{TeW_8\}$ fragments. The Ce5–O bond lengths vary from 2.474 (17) to 2.568 (16) Å.

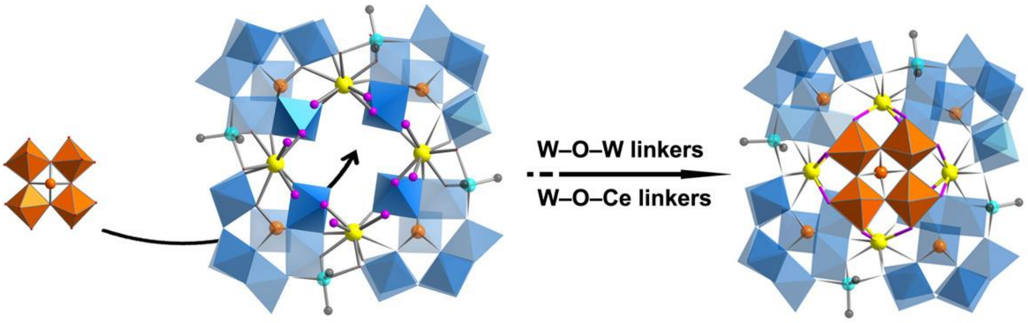

Figure S7. The combination of $\{Sn_4Ce_4(TeW_8)_4\}$ and $\{TeW_4\}$ segments through W–O–W and W–O–Ce bonds, in which the bridging O atoms are highlighted in purple color and W–O–W and W–Ce–O bonds are highlighted in two-colored style.

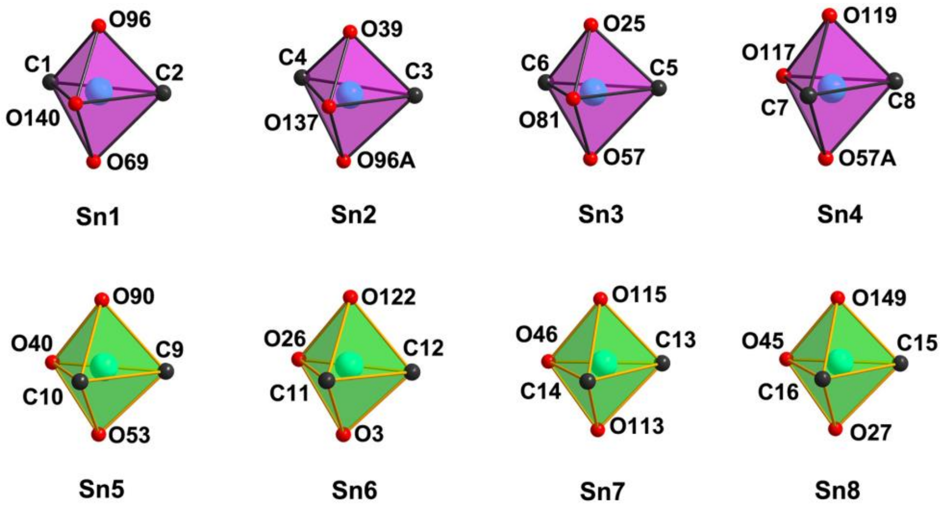

Figure S8. The coordination spheres and coordination configurations of Sn^{4+} ions in **1**.

There exist eight crystallographically unique Sn^{4+} ions in **1**. The Sn1–Sn4 ions in the dimerized $\{[Sn(CH_3)_2]_2O\}^{2+}$ segments display the penta-coordinate trigonal bipyramidal geometries architected from two C atoms from two $–CH_3$ groups and three O atoms from one $\{TeW_8\}$ fragment. The Sn–O/C bond lengthes are in the range of 1.997 (16)–2.254 (18) Å (Sn1), 1.987 (18)–2.182 (18) Å (Sn2), 2.002 (18)–2.226 (18) Å (Sn3) and 1.980 (18)–2.230 (18) Å (Sn4), respectively. The Sn5–Sn8 ions also show the penta-coordinate trigonal bipyramidal geometries, but the coordination environments are somewhat different. The trigonal bipyramid of Sn5–Sn8 ions is established by two C atoms from two $–CH_3$ groups, three terminal O atoms from two $\{TeW_8\}$ fragments. The Sn–O/C bond lengthes are in the range of 2.020 (17)–2.324 (16) Å (Sn5), 2.034 (16)–2.344 (17) Å (Sn6), 2.054 (18)–2.320 (16) Å (Sn7) and 2.053 (17)–2.351 (14) Å (Sn8), respectively. Symmetry operation: A: –x, y, 0.5–z.

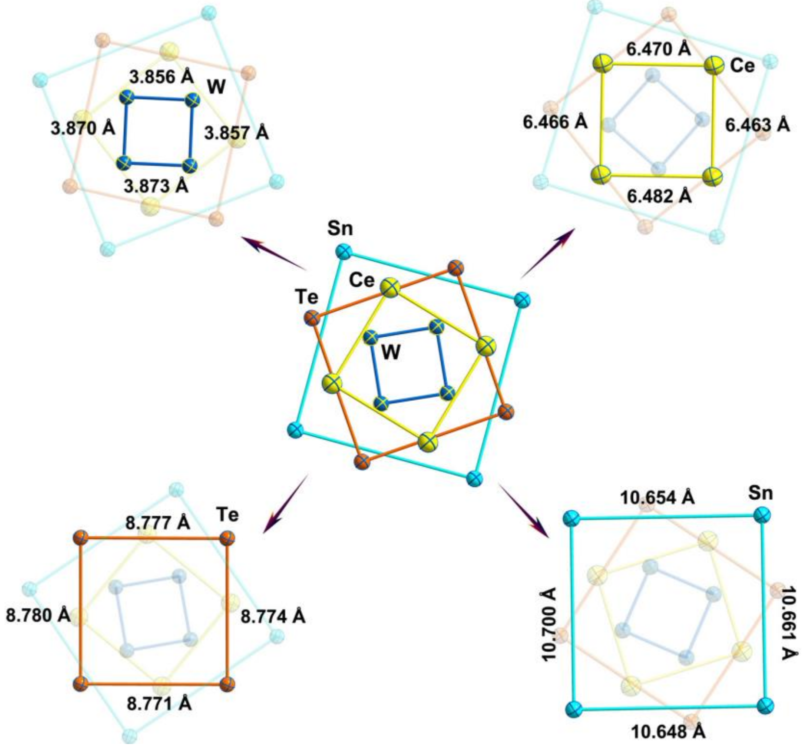

Figure S9. The square-planar multilayer arrangements of four inner W atoms, four Ce atoms, the outer four Te heteroatoms and four Sn atoms with variable sizes.

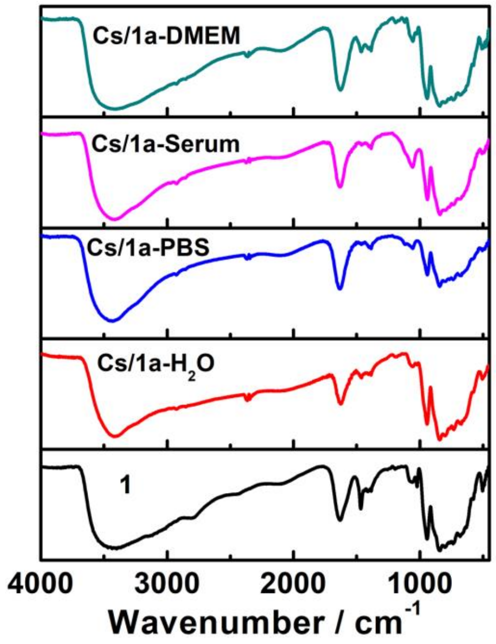

Figure S10. Comparison of IR spectra of **1**, Cs/**1a**-water, Cs/**1a**-PBS (PBS: 0.2 M, pH = 7), Cs/**1a**-Serum (serum diluted with 0.2 M PBS) and Cs/**1a**-DMEM precipitates (from bottom to top).

We used the method of precipitating **1a** from the solution by adding cesium chloride to assess the stability of **1a** under the conditions of the biological tests. This is based on the suggestion that if **1a** has disintegrated into smaller fragments in solution, precipitation of **1a** from solution

will not be possible, and any precipitation formed would show different spectral characteristics. To this end, **1** was dissolved in water and the addition of excess cesium chloride led to the formation of the Cs/**1a**-water precipitate. As shown in Figure S10, the IR spectrum of the Cs/**1a** precipitate formed in water is virtually identical to the original sample of solid **1**. Specifically, three groups of characteristic peaks at 942; 845, 799, 763; 681 cm^{-1} resulting from the v(W–O$_t$), v(W–O$_b$), and v(W–O$_c$) stretching vibrations of the Cs/**1a**-water precipitate is very near to those (942; 847, 805, 762; and 683 cm^{-1}) of **1**. Also, the band centered at 637 cm^{-1} ascribed to the v(Sn–O–Sn) vibration can be observed in the IR spectrum of the Cs/**1a**-water precipitate. This observation suggests that **1a** is stable in water. Similarly, **1** was dissolved in PBS, Serum and DMEM and the addition of excess cesium chloride led to the formation of Cs/**1a**-PBS**,** Cs/**1a**-Serum, and Cs/**1a**-DMEM precipitates. As shown in Figure S10, three groups of characteristic v(W–O$_t$), v(W–O$_b$), and v(W–O$_c$) peaks and v(Sn–O–Sn) vibration peak appear at 940; 845, 804, 765; 679 cm^{-1}; 737 cm^{-1}; 633 cm^{-1} for the Cs/**1a**-PBS precipitate, 940; 841, 801, 765; 682 cm^{-1}; 736 cm^{-1}; 633 cm^{-1} for the Cs/**1a**-Serum precipitate, and 941; 845, 800, 763; 681 cm^{-1}; 734 cm^{-1} and 632 cm^{-1} for the Cs/**1a**-DMEM precipitate, which is virtually identical to the characteristic signals of solid **1**. These results further support that **1a** is stable under the conditions of the biological tests. In addition, the variations of the stability of **1a** in water, PBS, Serum and DMEM with time have been measured by Raman. As shown in Figure S11, the characteristic signals of **1a** are retained for 48 h in water, PBS, Serum and DMEM.

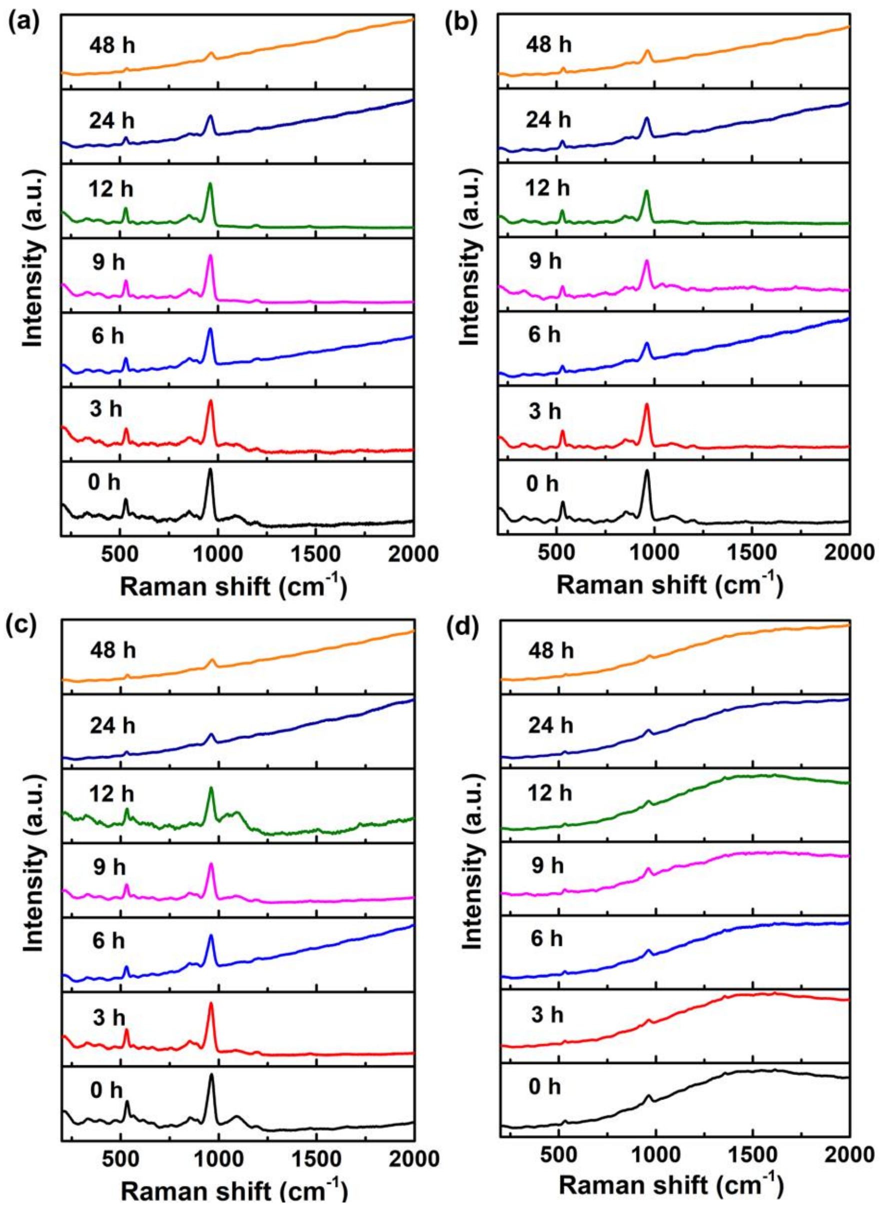

Figure S11. Raman spectra of **1** in (a) H_2O, (b) PBS (0.2 M, pH = 7), (c) Serum (diluted by 0.2 M PBS) and (d) DMEM with laser excitation at 530 nm. Note that the low signal intensity in (d) is most likely due to low solubility of **1**.

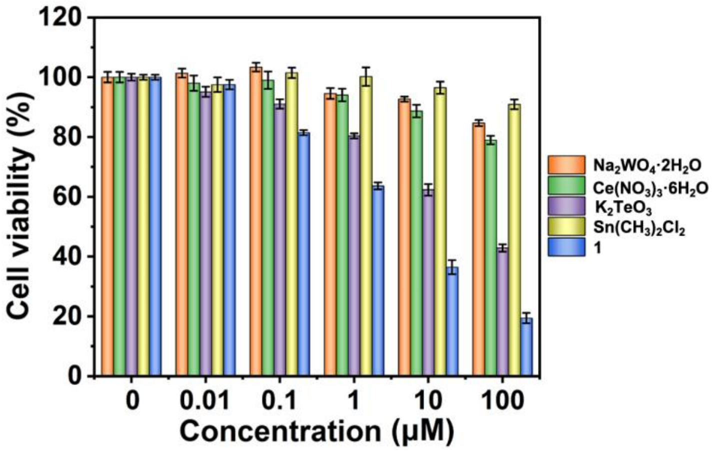

Figure S12. Cytotoxicity of HeLa cells resulting from a 24 h treatment. HeLa cells were incubated with $Na_2WO_4·2H_2O$, $Ce(NO_3)_3·6H_2O$, K_2TeO_3, $Sn(CH_3)_2Cl_2$ and **1**. The data are presented as mean ± S.D. from three parallel experiments.

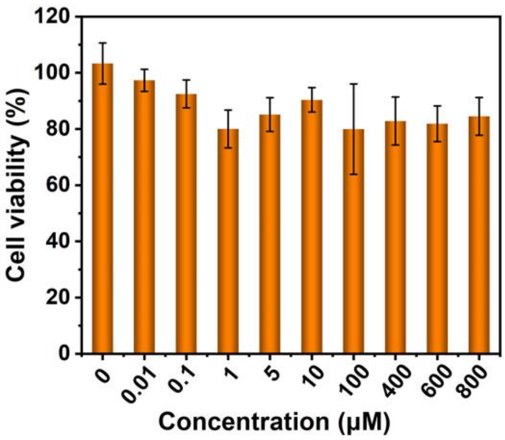

Figure S13. Cytotoxicity of 5-FU on HeLa cells resulting from a 24 h treatment. The data are presented as mean ± S.D. from three parallel experiments.

MTT analysis of 5-FU toward HeLa cells has been studied, and the cell viability is higher than 81.9% with concentrations of 5-FU in the range of 0.1–800 μM (Fig. S13), suggesting that **1** shows higher cytotoxic performance toward HeLa cells than 5-FU (both in terms of molar and weight concentration). This is true for both molar and weight concentrations: MTT results show that **1** reaches a viability of 61.0% at a concentration of 60.34 μg/mL (2 μM), while 5-FU showed a viability of 81.9 % at a concentration of 78.04 μg/mL (600 μM).

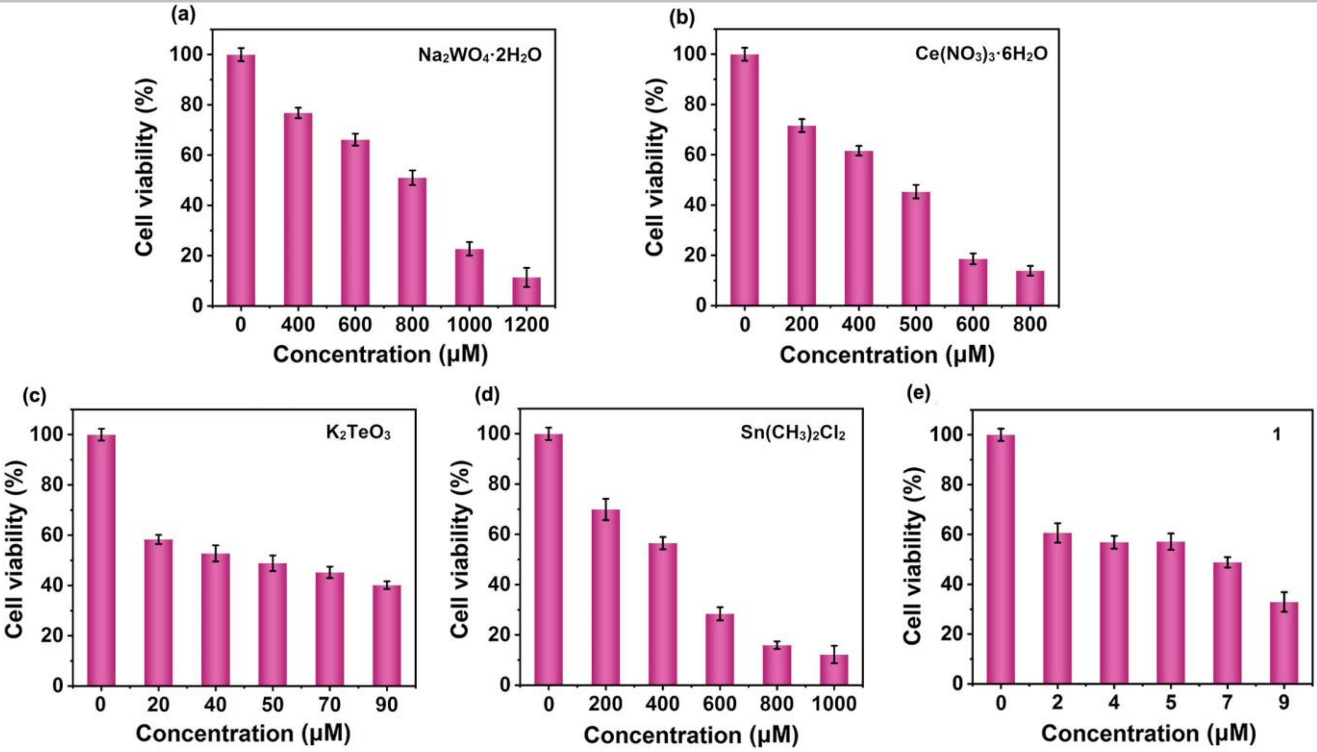

Figure S14. Cytotoxicity of HeLa cells resulting from a 24 h treatment of (a) $Na_2WO_4{\cdot}2H_2O$; (b) $Ce(NO_3)_3{\cdot}6H_2O$; (c) $Sn(CH_3)_2Cl_2$; (d) K_2TeO_3 and (e) **1**. The data are presented as mean ± S.D. from three parallel experiments.

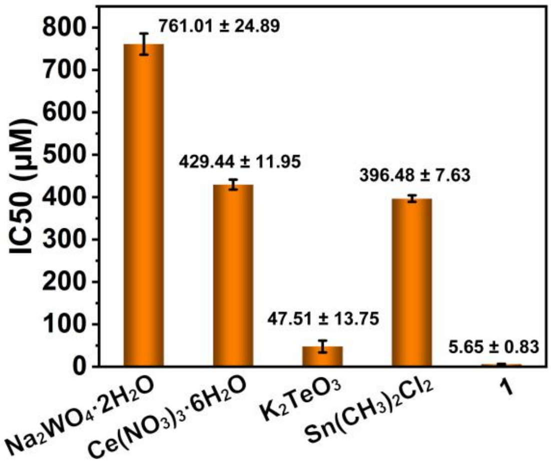

Figure S15. Comparison of IC_{50} values of $Na_2WO_4{\cdot}2H_2O$, $Ce(NO_3)_3{\cdot}6H_2O$, K_2TeO_3, $Sn(CH_3)_2Cl_2$ and **1** against HeLa cells. The data are presented as mean ± S.D. from three parallel experiments.

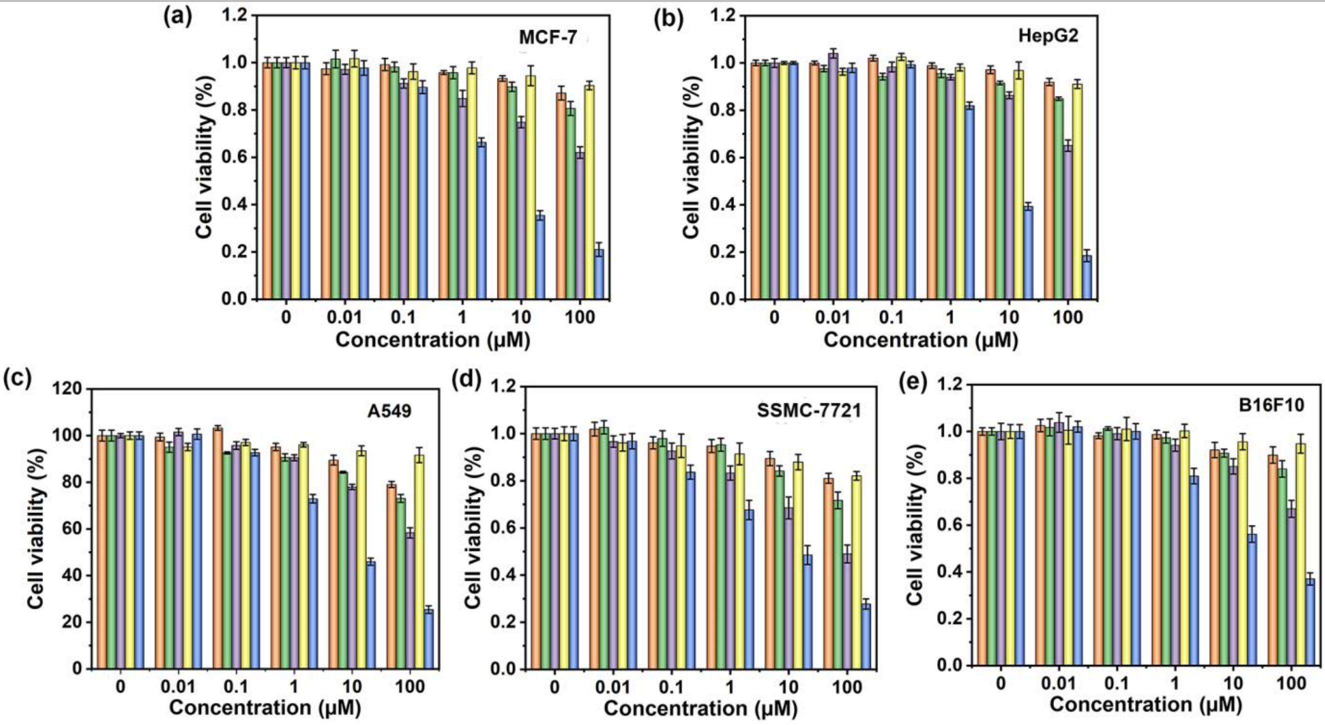

Figure S16. Cytotoxicity for MCF-7, HepG2, A549, SMMC-7721 and B16F10 cells resulting from a 24 h treatment. These cells were incubated with $Na_2WO_4{\cdot}2H_2O$ (orange) , $Ce(NO_3)_3{\cdot}6H_2O$ (green), K_2TeO_3 (purple), $Sn(CH_3)_2Cl_2$ (yellow) and **1** (blue). The data are presented as mean ± S.D. from three parallel experiments.

Cytotoxicity against MCF-7, HepG2, A549, SMMC-7721 and B16F10 cells was assessed using MTT assays after the cells were incubated with **1** (0.01, 0.1, 1.0, 10.0 and 100.0 μM) for 24 h. Experimental results show that **1** inhibits the cell proliferation in a dose-dependent manner, and the viabilities of MCF-7, HepG2, A549, SMMC-7721 and B16F10 cells treated with 100.0 μM **1** at 24 h decrease to 21.06%, 18.51%, 25.44%, 27.73% and 37.02%, respectively, which reveals an cytotoxic effect.

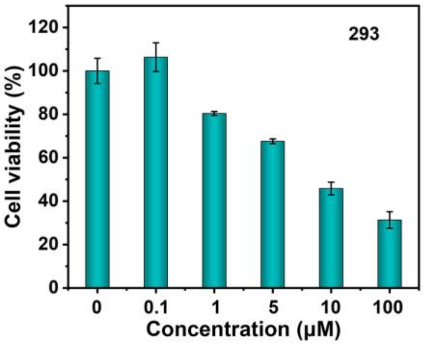

Figure S17. Cytotoxicity of 1 towards kidney epithelial 293 cells resulting from a 24 h treatment with **1**. The data are presented as mean ± S.D. from three parallel experiments.

Cytotoxicity against kidney epithelial 293 cells was also evaluated by MTT assays. The cells were incubated with **1** (0.1, 1.0, 5.0, 10.0 and 100.0 μM) for 24 h, and the viability decreases to 31.33% at a concentration of **1** of 100 μM. This result indicates that **1** also shows cytotoxicity to non-tumoral cells such as kidney epithelial cell lines, although cytotoxicity in this case is marginally lower compared with the cytotoxicity observed for HeLa cells.

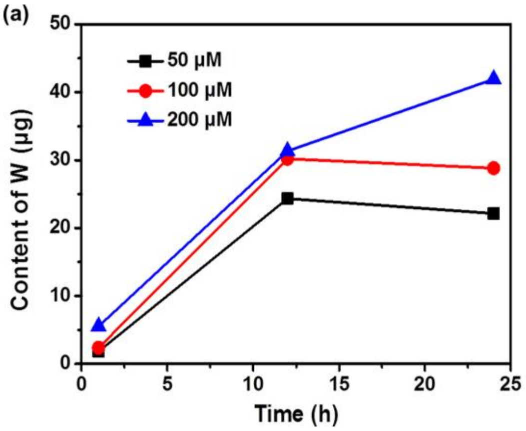

Figure S18. (a) Time- and concentration-dependent tungsten uptake by HeLa cells after incubation with different concentrations of **1**

Cellular uptake tests were performed after incubation of HeLa cells with different concentrations of **1** for different times; W content was determined by ICP analyses as follows: HeLa cells were cultured at 37 ºC for 24 h, then adding 10 mL of aqueous solutions of **1** (50, 100, 200 μM) and incubated for different times. The cells were digested, rinsed with PBS three times, and collected. Then, cells were treated with 4 mL HNO_3 to lyse cells for ICP test. As shown in Figure S18, under the same incubation times of 1, 12 and 24 h, the W content increases with increasing the concentration of **1** from 50 to 100 to 200 μM.

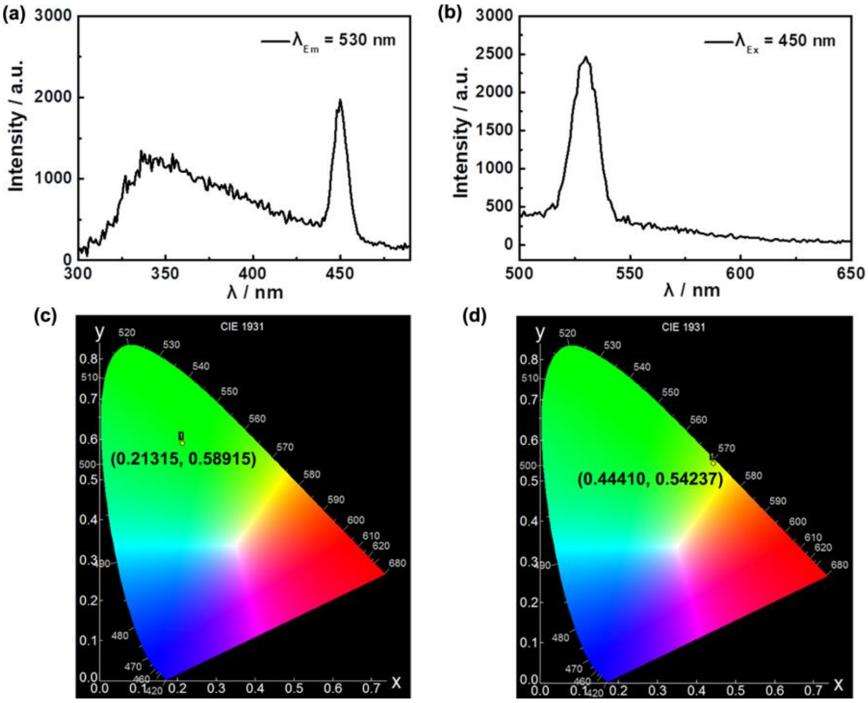

Figure S19. (a) Excitation spectrum of **1** in aqueous solution (100 μM). (b) Emission spectrum of **1** in aqueous solution (100 μM) at an excitation wavelength of 450 nm. (c) Emitting color of **1** in the CIE diagram (λ_{Ex} = 450 nm, λ_{Em} = 530 nm). (d) Emitting color of **1** in the CIE diagram (λ_{Ex} = 488 nm, λ_{Em} = 564 nm).

Confocal fluorescence images of HeLa cells treated with **1** were collected to observe the uptake of **1** by HeLa cells. Emission spectroscopy was first used to identify suitable excitation and emission bands for analyzing **1**. Based on this analysis, excitation at 488 nm was chosen for confocal fluorescence imaging (leading to an emission maximum at 564 nm). Confocal fluorescence imaging was

performed after incubating HeLa cells for 12 h in 100 μM **1**. The data show an enhanced emission signal (enhancement factor *ca.* 1.4) when compared with the reference sample (Fig. S20/S21).

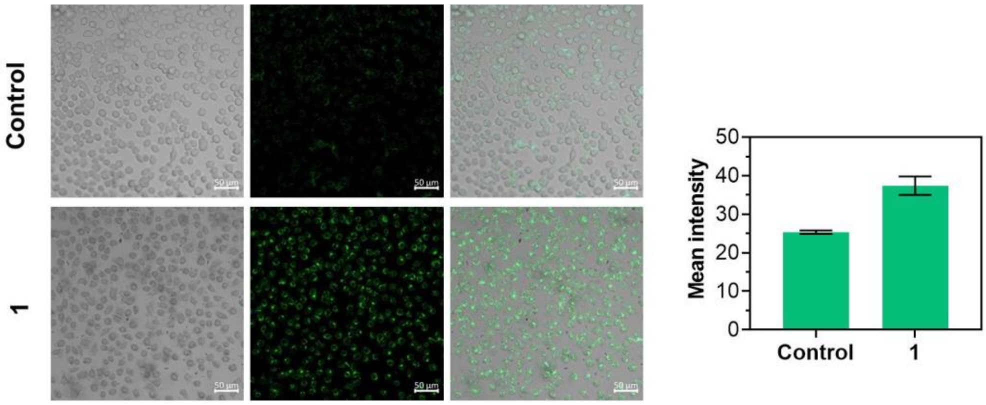

Figure S20. Confocal fluorescence images of HeLa cells incubated with **1** for 12 h and their intensity comparison (data are presented as mean ± S.D. from seven experiments).

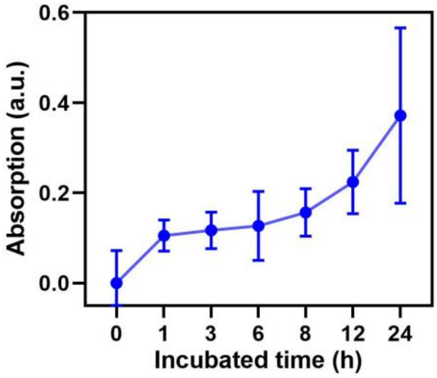

Figure S21. The change of the absorption intensity with incubation time. The data are presented as mean ± S.D. from three parallel experiments. Absorbance spectrophotometry was measured at 250 nm (using a characteristic absorption of **1**) using a microplate reader (SpectraMaxi3x).

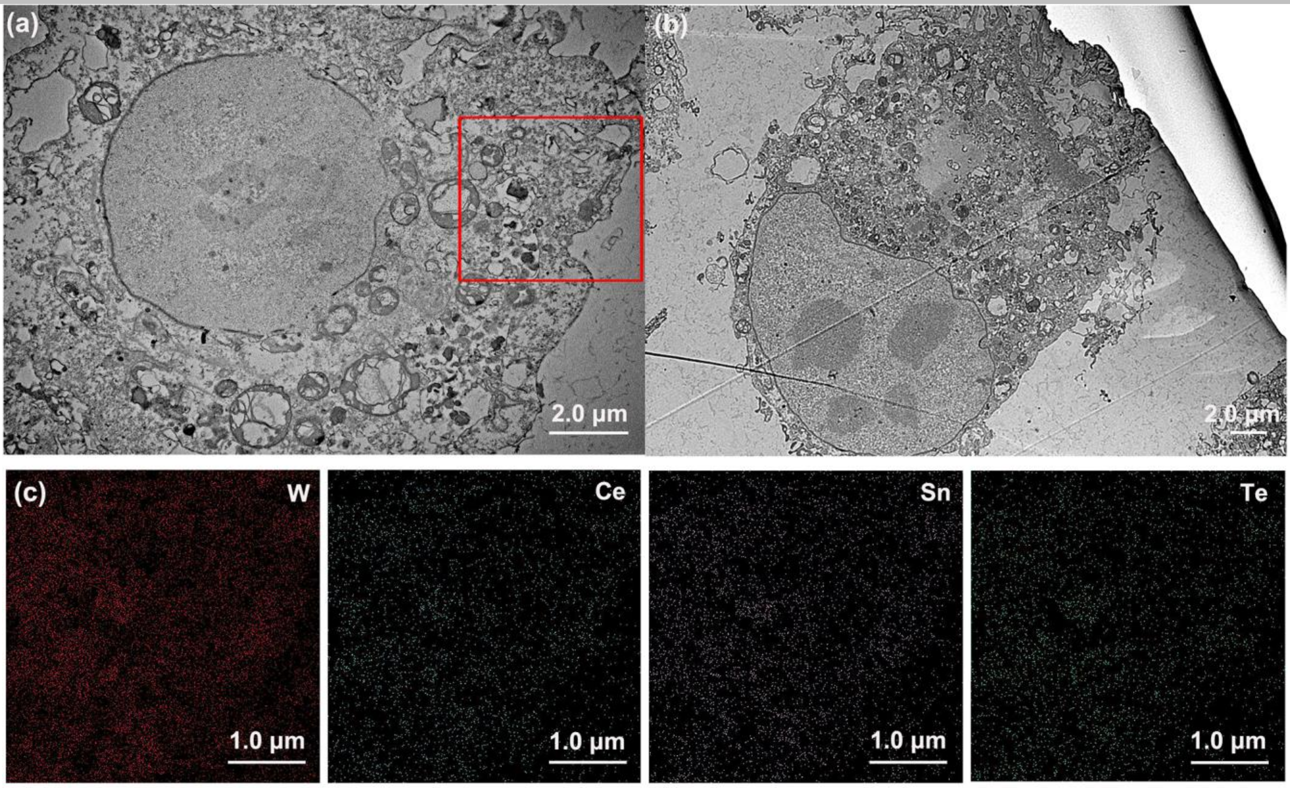

Figure S22. (a) and (b) Bio-TEM images of HeLa cells incubated with 100 μM **1** for 24 h. (c) Elemental mappings of the corresponding circled region in (a).

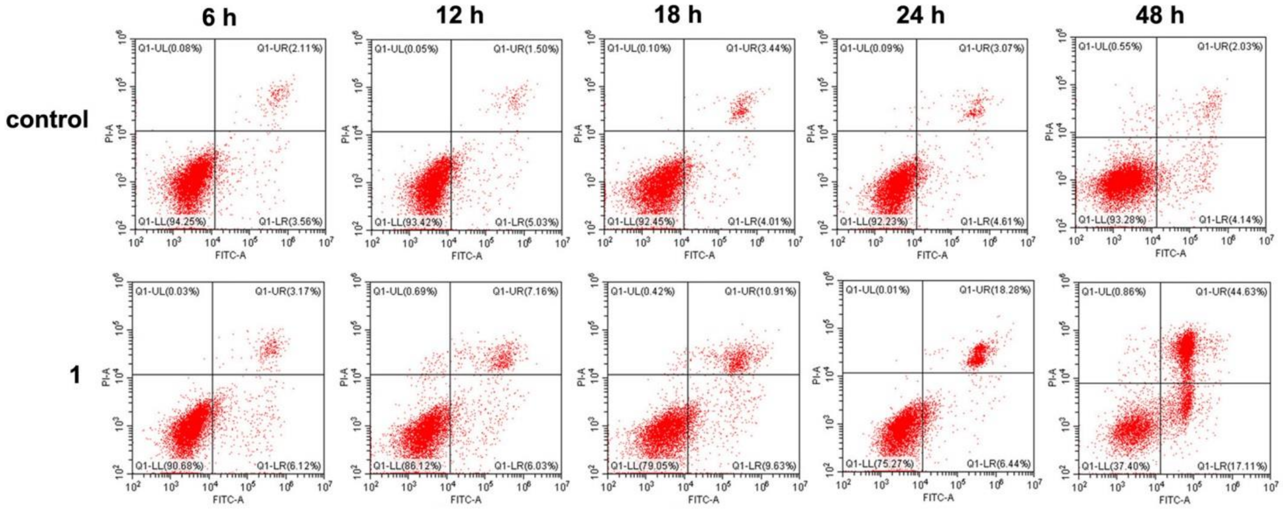

Figure S23. Flow cytometry analysis of apoptosis. Cells were untreated, treated with 3 μM **1** for 6, 12, 18, 24 and 48 h and stained with Annexin V-FITC-PI. Representative pictures shown from three parallel experiments.

The annexin V-FITC together with propidium (PI) assay are generally utilized to distinguish viable, apoptotic and necrotic cells that could not be examined in MTT assay. Viable cells that aren't stained with Annexin V-FITC or PI are located at Q1-LL quadrant. While apoptotic

cells move to the right after binding with Annexin V-FITC and PI, in which early and advanced apoptotic cells are situated at Q1-LR and Q1-UR quadrants.

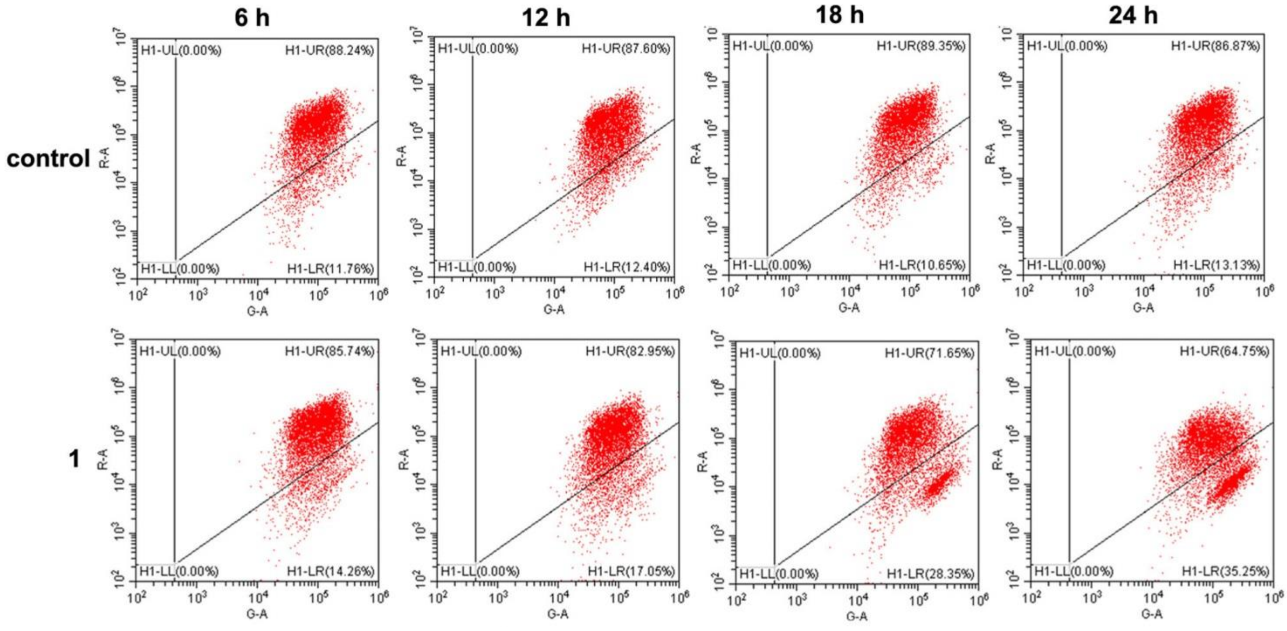

Figure S24. Effect of untreated, treated with 3 μM **1** on the MMP in HeLa cells under various times of 6, 12, 18 and 24 h. Representative pictures shown from three parallel experiments. The change of MMP is also an important symbol to evaluate cell apoptosis.

The release of apoptotic factors such as cytochrome C or apoptosis-inducing factor would cause the decrease of MMP and tigger cell apoptosis.[7] The MMP change can be detected by staining live cells with JC-1 cationic fluorescent dye, as demonstrated by the altering of fluorescence signal from red to green upon decreasing MMP.[8−9] Here, the JC-1 red/green signal ratio was calculated by the H1-UR/H1-LR ratio.

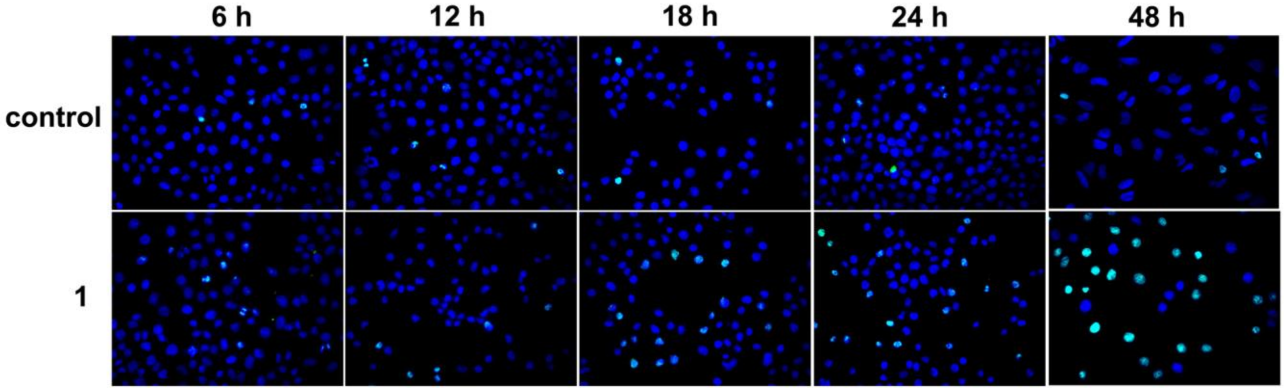

Figure S25. TUNEL staining for 6, 12, 18, 24 and 48 h. Representative pictures shown from three parallel experiments.

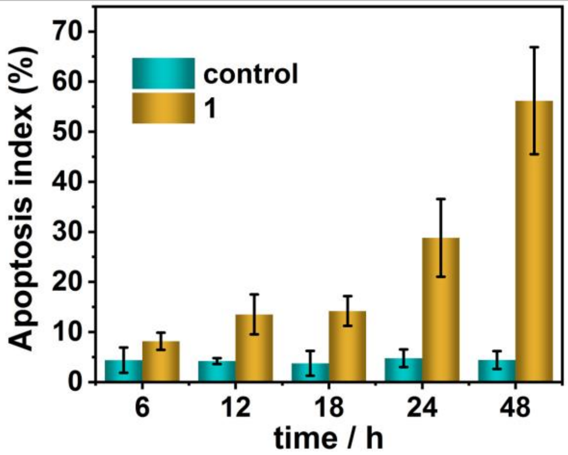

Figure S26. Change of the apoptosis index along with time.

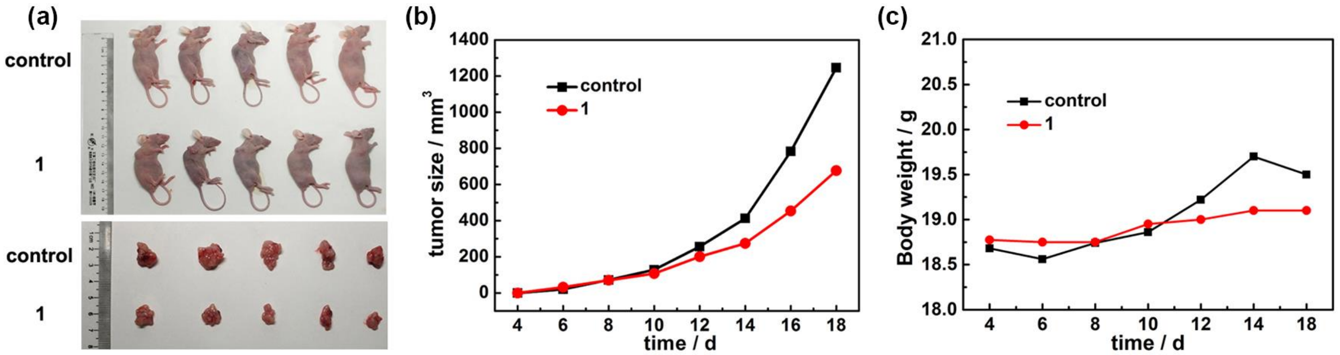

Figure S27. (a) *In vivo* experiments on mice and photographs of excised tumors. (b) Growth of tumor size in mice. (c) Body weights of HeLa-untreated and HeLa-bearing nude mice.

The tumor sizes and the weight of nude mice were measured. For the **1**-treated mice, an average tumor volume of 677 mm^3 was observed, while the control group showed an average volume of 1247 mm^3 at the 18th day, indicating **1** can inhibit the tumor progression. Besides, no obvious changes in weight (the average weight ranges from 18.75 to 19.10 g) indicate that there is no noticeable side effect of **1** on the mice.

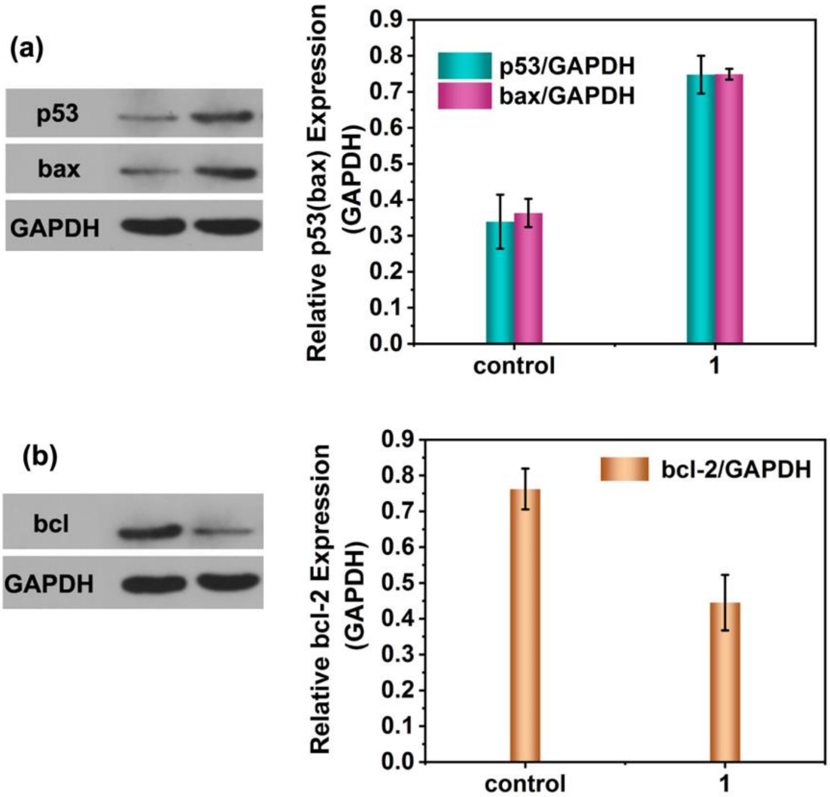

Figure S28. (a) and (b) Expression of p53, bax and bcl-2 in HeLa cells determined by WB analyses. GAPDH was used as the loading control. The control cells were untreated, the sample cells were treated with 3 µM **1**.

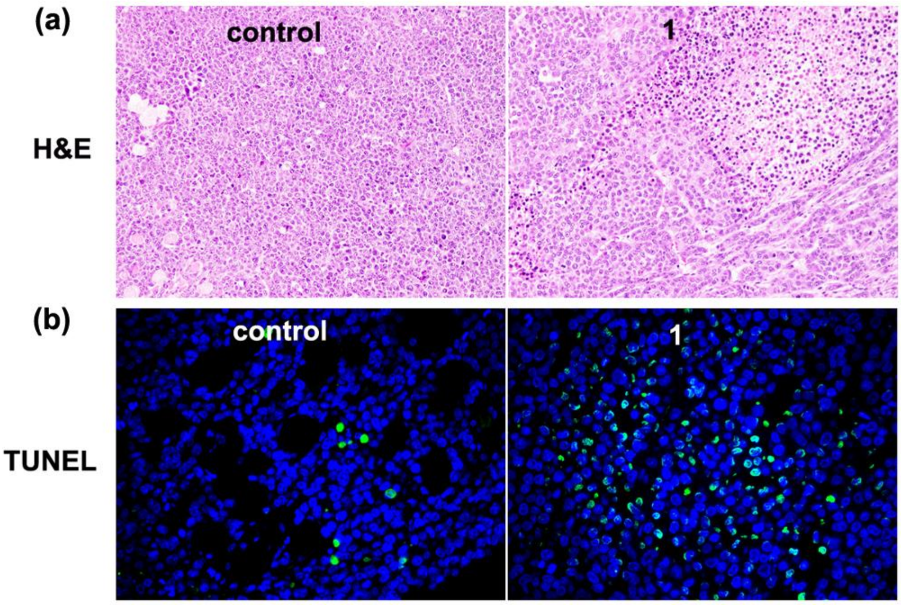

Figure S29. (a) and (b) The H&E staining and TUNEL assay of tumor slices from mice untreated and treated with **1**. Concentration: 3 µM **1**.

S4 Bond valence sum (BVS) calculations

Table S2. Bond valence sum (BVS) calculations of Ce, Te and W atoms in **1**.

Atom	BVS	Atom	BVS	Atom	BVS	Atom	BVS	Atom	BVS
Ce1[10]	2.97	W1[11]	6.23	W11	6.00	W21	6.11	W31	6.06
Ce2	2.91	W2	6.26	W12	6.25	W22	6.15	W32	6.00
Ce3	2.90	W3	6.31	W13	6.32	W23	6.09	W33	6.06
Ce4	2.88	W4	6.10	W14	6.27	W24	5.88	W34	6.14
Ce5	2.73	W5	5.88	W15	6.16	W25	6.07	W35	6.01
Te1[11]	4.01	W6	6.06	W16	6.03	W26	5.88	W36	6.12
Te2	3.78	W7	6.04	W17	6.15	W27	5.86	W37	6.54
Te3	4.06	W8	6.04	W18	5.97	W28	6.42	W38	6.58
Te4	3.92	W9	6.27	W19	6.03	W29	6.19	W39	6.21
Te5	4.14	W10	6.47	W20	6.46	W30	5.85	W40	6.09
								W41	6.06

S5 References

[1] Q. Liu, H. W. Dong, W. G. Sun, M. Liu, J. C. Ibla, L. X. Liu, J. W. Parry, X. H. Han, M. S. Li, J. R. Liu, *Arch. Toxicol.* **2013**, *87*, 481–490.

[2] G. M. Sheldrick, *SADABS: Program for Empirical Absorption Correction of Area Detector Data*; University of Göttingen, Germany, **1996**.

[3] (a) G. M. Sheldrick, *SHELXS 97, Program for Crystal Structure Solution*; University of Göttingen, Germany, **1997**; (b) G. M. Sheldrick, *SHELXL 97, Program for Crystal Structure Refinement*, University of Göttingen, Germany, **1997**.

[4] S. Reinoso, M. H. Dickman, U. Kortz, *Inorg.Chem.* **2006**, *45,* 10422–10424.

[5] Q. Han, J.-C. Liu, Y. Wen, L.-J. Chen, J.-W. Zhao, G.-Y. Yang, *Inorg. Chem.* **2017**, *56,* 7257–7269.

[6] L. Zhang, K. N. Zhao, R. H. Yu, M. Y. Yan, W. W. Xu, Y. F. Dong, W. H. Ren, X. Xu, C. J. Tang, L. Q. Mai, *Small* **2017**, *13,* 1603973.

[7] S. A. Susin, H. K. Lorenzo, N. Zamzami, I. Marzo, B. E. Snow, G. M. Brothers, J. Mangion, E. Jacotot, P. Costantini, M. Loeffler, *Nature* **1999**, *397*, 441–446.

[8] C. X. Wang, R. J. Youle, *Annu. Rev. Genet*. **2009**, *43*, 95–118.

[9] C. P. Tan, S. S. Lai, S. H. Wu, S. Hu, L. J. Zhou, Y. Chen, M. X. Wang, Y. P. Zhu, *J. Med. Chem.* **2010**, *53*, 7613–7624.

[10] A. Trzesowska, R. Kruszynski, T. J. Bartczak, *Acta Cryst*. **2004**, *B60*, 174–178.

[11] I. D. Brown, D. Altermatt, *Acta Cryst*. **1985**, *B41*, 244–247.
